# Quality indicators and community pharmacy services: a scoping review

**DOI:** 10.1111/ijpp.12561

**Published:** 2019-07-02

**Authors:** Nour Alhusein, Margaret C. Watson

**Affiliations:** ^1^ Department of Pharmacy and Pharmacology University of Bath Bath UK; ^2^ Watson Research and Training Limited Aberdeen UK

**Keywords:** healthcare quality indicators, pharmaceutical services, pharmacies, pharmacists, quality improvement, quality of health care

## Abstract

**Background:**

Quality indicators are a commonly used improvement tool in health care. There is growing interest and activity in the use of quality indicators to improve community pharmacy practice.

**Objectives:**

To conduct a scoping review of the use of quality indicators for community pharmacy practice, including their methods of development and evaluation.

**Methods:**

Electronic databases (EMBASE and PubMed) were searched to identify papers published between January 2008 and April 2018. No limits were applied for language of publication or country of origin. Studies were included if they reported empirical data regarding the development or evaluation of quality indicators. All study designs were eligible for inclusion. Duplicate independent screening was undertaken of the search results. Data extraction was performed by one reviewer.

**Results:**

Of the 988 records identified from the database search, 15 articles were included. The studies were conducted in 12 countries from six continents. Eleven studies described the development of quality indicators, eight of which included the evaluation of the psychometric properties of the indicators developed. Four studies examined the impact of quality indicators on practice all of which reported improvements in some aspects of quality, mainly with structure indicators rather than those relating to process and outcome.

**Conclusions:**

Whilst there is a growing emphasis on promoting improvement in community pharmacy services, evidence is lacking of the effect of indicators on improving quality. Measurable process and outcome indicators are needed. The future development of quality indicators would also benefit from a multi‐stakeholder approach.

## Introduction

Many healthcare systems suffer from poor quality leading to preventable deaths, reduced quality of life or serious adverse events, such as medication errors.^1^, ^2^ Reports on failures in the quality of health care have called for healthcare reform and quality improvement.^2^, ^3^, ^4^

Quality and quality improvement are multi‐dimensional concepts.^5^ Quality improvement was defined as ‘the combined and unceasing efforts of everyone – healthcare professionals, patients and their families, researchers, payers, planners and educators – to make the changes that will lead to better patient outcomes (health), better system performance (care) and better professional development (learning)’.^6^

With this definition in mind, five knowledge systems have been recognised as being involved in improvement, including: generalisable scientific evidence; particular context awareness; performance measurement; plans for change; and execution of planned changes.^6^ One of these systems is performance measurement which includes the use of balanced measures that can assess the effect of changes in quality over time. Quality indicators are required to measure performance and are ‘measurable elements of practice performance for which there is evidence or consensus that it can be used to assess the quality, and hence change in the quality, of care provided’.^7^ Quality indicators, like many healthcare instruments, are often subject to a psychometric validation (e.g. validity, reliability, feasibility, sensitivity to change) to ensure their suitability for quality assessment. Psychometric validation assesses each instrument through a series of defined tests on the population group for whom the instrument is intended.^8^

There is growing pressure to demonstrate and improve the quality of health care delivered in community pharmacies.^9^, ^10^, ^11^, ^12^ This demand is partly driven by the need to determine and evidence how the extended role of community pharmacy teams contributes towards health service delivery and the reduction of pressure on other health sectors.^13^, ^14^, ^15^

In 1999, the International Pharmaceutical Federation (FIP) and the World Health Organization (WHO) published a joint document on good pharmacy practice in community and hospital pharmacy settings.^16^ The document encouraged national pharmaceutical organisations to direct pharmacists to ensure service provision of appropriate quality to every patient. The FIP provided support to its member organisations in different countries for example Cambodia, Mongolia, Paraguay and Thailand, to develop their own national standards in line with the recommendations of FIP/WHO.^17^

Other countries have invested in developing quality indicators in community pharmacy. For example, in Australia, The Quality Care Pharmacy Program was established to assure and improve quality in community pharmacies.^11^ In the United States,^18^ the Pharmacy Quality Alliance, a voluntary, membership‐based collaborative comprising organisations from pharmacy, patient, employer and the health insurance plan communities, as well as state and federal government, committed to develop quality measure concepts in community pharmacies. Similarly, since 2008 in the Netherlands,^19^ the Dutch Health Care Transparency Programme has been working on developing quality indicators and enhancing their uptake in everyday practice. More recently in the UK in 2016,^20^ eight quality indicators were introduced into the Community Pharmacy Contractual Framework with revised elements introduced for 2019.^21^ The domains to which the payments apply included patient safety, public health, clinical effectiveness, digital/urgent care and the workforce. There is often limited information regarding how quality indicators have been developed, who participated in producing them and their impact on practice and patients outcomes.

A widely accepted conceptual framework to assess the quality of medical care or healthcare services was described by Donabedian.^22^, ^23^ The framework consists of three components in which quality indicators can be classified: structure, process and outcome. Structure indicators refer to the setting and the resources in which the care occurs, such as medical supplies, vehicles, personnel and organisational structure. Process indicators relate to interactions and what is actually done when giving and receiving care. Outcome indicators are associated with the consequences for the health status of patients or the population for example patient satisfaction. Standardised indicators and tools are needed to measure and improve the quality of community pharmacy services. A lack of appropriate indicators and tools may contribute to inconsistency between pharmacies and the quality of care delivered.^24^, ^25^ A recent study from the UK highlighted inconsistencies in community pharmacists’ attitudes towards, and beliefs about, quality in terms of how it is defined and measured.^26^

The aim of this study was to undertake a scoping review of studies which reported the development and/or evaluation of quality indicators for use in community pharmacies. The objectives were to explore how quality indicators were developed, by whom and the methods of evaluation used to assess their effect on practice.

## Methods

A scoping review was undertaken to identify studies that had developed or evaluated quality indicators in community pharmacy settings. (The review protocol can be accessed on request from the authors.) This review was conducted to comply with the PRISMA‐ScR statement for scoping reviews.^27^

### Search strategy

Electronic databases (EMBASE and PubMed) were searched from January 2008 to April 2018 using a combination of keywords, Emtree (EMBASE) and/or Medical Subject Headings (MeSH) (PubMed) (Appendix S1). Due to limited resources, the grey literature was not searched and search terms were limited to ‘major’ domains within the research databases. The concept of quality indicators is relatively new to community pharmacy; hence, the literature search was restricted to the period from January 2008. Additional studies were identified by searching the reference lists of retrieved articles and the authors’ reference collections.

### Study selection process

The results from the electronic databases were imported into EndNote (version 8; Clarivate, Philadelphia, Pennsylvania USA), and duplicate records were removed. Duplicate independent screening of the titles and abstracts was undertaken to identify records which appeared to fulfil the inclusion criteria. The full‐text versions of potentially eligible records were retrieved and assessed independently in duplicate against the inclusion criteria.

### Inclusion criteria

Studies were included if they reported the development of quality indicators for community pharmacy practice or evaluated the effect of quality indicators on practice. No limits were set on the language of publication or by the country of origin. Non‐English records were translated using Google Translate. When the accuracy of the translation was unclear, a bilingual person was consulted. Studies were included if they presented empirical data (qualitative and/or quantitative). All study designs were eligible for inclusion.

### Exclusion criteria

Studies were excluded if they were as follows: poster abstracts, commentaries, literature reviews, assessed the quality of a single service (e.g. medicine use review), assessed only one element of quality (e.g. safety consumer satisfaction) and/or implemented or assessed the feasibility of using a quality data collection software (e.g. online data collection platform). We focused upon the development and testing of QIs using a holistic approach, that is whole sets of QIs, rather than reporting on one aspect of quality only. National quality indicator programmes were eligible for inclusion only if their development or evaluation was reported.

### Data extraction

A bespoke data extraction form was developed, and data extraction was undertaken by one researcher (NA). Data extraction included: article characteristics (year of publication, country of origin and funders), study aims, participants, quality indicator characteristics, study design and methods, analysis and results, and strength and limitations (Appendix S2). Due to the heterogeneity of the studies included, the results are presented as a narrative synthesis.

## Results

### Literature search and studies characteristics

**Figure 1:**
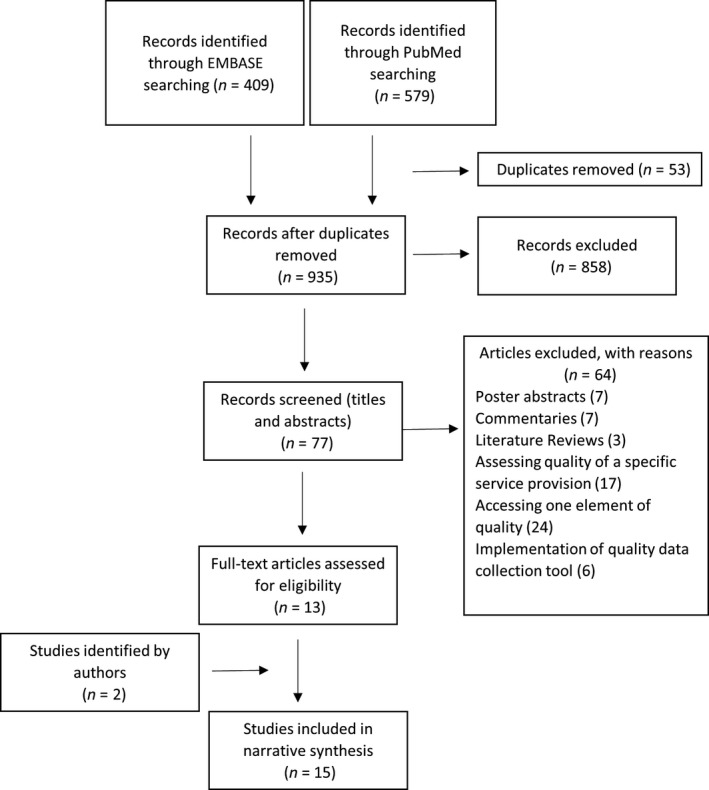
PRISMA flow chart of study selection process

In total, 15 articles were identified as meeting the inclusion criteria. Figure 1 illustrates the study selection and the exclusion/inclusion processes. The studies were conducted in 12 countries, of which three were conducted in the Netherlands,^28^, ^29^, ^30^ two each in the UK,^31^, ^32^ USA,^33^, ^34^ and Thailand,^35^, ^36^ and one each in Argentina,^37^ Spain,^38^ Australia,^39^ Canada^40^ and Brazil.^41^ One study^42^ was conducted in three countries: Ethiopia, Uganda and Zimbabwe. Two studies were published in Spanish,^37^, ^38^ one in Portuguese^41^ and one in Thai.^35^ The remainder were published in English.

### The development and psychometric testing of quality indicators

Eleven studies reported the development of quality indicators for community pharmacies.^28^, ^29^, ^31^, ^32^, ^33^, ^34^, ^35^, ^36^, ^37^, ^40^, ^42^ Multiple methods were used to produce initial sets of indicators including literature review,^28^, ^37^, ^42^ focus groups,^31^, ^33^ surveys,^32^, ^37^ case studies^32^, ^43^ and interviews^33^ (Table 1). Initial sets of indicators were subjected to a selection exercise performed by expert or stakeholder consensus.^28^, ^32^, ^33^, ^37^ Testing of psychometric properties included validity,^28^, ^29^, ^33^, ^37^ reliability,^29^, ^33^, ^35^ feasibility^28^, ^34^, ^36^, ^37^, ^40^ and variability.^33^

**Table 1:** Studies reporting the development of quality indicators

First author Country year	Steps involved in developing quality indicators	Participants	Psychometric characteristics sought in the study
Grey^32^ UK 2016	Delphi‐style survey (two rounds)	22 participants completed both Delphi rounds Dispensing GPs (*n* = 2), community pharmacists (*n* = 8), pharmacy dispensing assistants (*n* = 2), pharmacy organisation board members (*n* = 1), large chain community pharmacy executives (*n* = 2), patients (*n* = 7)	
Wongpratat^35^ and Arkaravichien^36^ Thailand 2015, 2016	The tool was originally developed by the Community Pharmacy Association, with technical support from the FIP		Reliability
	Observation and interviews with pharmacists to collect data on the QI set	Accredited (*n* = 30) and non‐accredited pharmacies (*n* = 30), which were paired to the nearest setting of the accredited ones (500 m radius) in the north‐east region	
	Observation and interviews with pharmacists to collect data on the QI set	81.1% (*n* = 60) of all accredited pharmacies in the north‐eastern part of Thailand	Feasibility
Schoenmakers^29^ Netherlands 2015	Online questionnaire provided data on the QI set collected in all Dutch community pharmacies	91% (*n* = 1807) of all community pharmacies in the Netherlands in 2011	Content validity Absence of selection bias Absence of measurement bias Statistical reliability
	Expert panel consensus	Expert panel of six pharmacists: practicing community pharmacists (*n* = 5) and a pharmacist/epidemiologist (project leader)	
Blalock^33^ USA 2012	Literature review		Internal consistency Reliability Construct validity Variability
	Focus groups	Four focus groups of consumers of pharmacy services (*n* = 30)	
	Interviews	Interviews with pharmacy patients (*n* = 12)	
	Stakeholders feedback and consensus	Pharmacy experts’ feedback (*n* > 50)	
	Survey/field test	Patients (*n* = 895)	
Halsall^31^ UK 2012	Focus group	47 in total, patients and carers (*n* = 21), health managers (*n* = 16), and pharmacists and pharmacy staff (*n* = 10).	
Winslade^40^ Canada 2011	Feasibility of routine medication‐related information of patients to screen the quality of care provided at community pharmacies.	Quebec's medication insurance programme provided data on prescriptions dispensed in 2002 by more than 5000 pharmacists in 1799 community pharmacies (*n* = 1.4 million patients)	Feasibility
Bie^28^ Netherlands 2011	Literature and guideline search followed by two consensus rounds	Working group, pharmacy practice experts (round 1) (*n* = 14) and practising pharmacists in community (round 2) (*n* = 76)	Face validity for quality of care or risk of harm to patients Clarity of wording Feasibility of data collection.
Trap^42^ Ethiopia, Uganda and Zimbabwe 2010	Previous literature and different policies		
	Field test: direct observations, record reviews, interviews and simulated clients visits	32 private and 39 public facilities in Ethiopia, 27 private and 33 public in Zimbabwe and 33 private in Uganda	
	Field test/ feasibility of data collection	Pharmacies (*n* = 30)	Feasibility
Pillittere‐Dugan^34^ USA 2009	Observational cohort study of pharmacy claim data of patients served by cross‐sectional of pharmacies from four health plans	Plan A (*n* = 850, 461) Plan B (*n* = 867, 016) Plan C (*n* = 35, 369) Plan D (*n* = 1, 185)	Feasibility of creating measures for each concept area of quality using only prescription drug claims data
Sevilla^37^ Argentina 2008	Literature review of existing quality frameworks around the world		Face and content validity, Feasibility
	Interviews	Pharmaceutical professionals and relevant official	
	Consensus using a nominal group technique	Pharmacists with recognised professional experience and teachers with experience in the development of accreditation programmes	

In Argentina,^37^ a study was conducted to provide tools for accreditation of community pharmacies including evaluation components and quality indicators. The study used interviews with pharmaceutical professionals and official bodies, which explored quality criteria for health care in relation to international trends and recommendations, as well as Donabedian’s three dimensions (structure, process and outcome).^22^, ^23^ A nominal group technique^30^ was used to derive consensus on the criteria based on evaluating the face and content validity, the feasibility and the importance of the indicators. The process produced 24 quality indicators which included three structures (documentation *n* = 2, equipment/supplies *n* = 4 and human resources *n* = 4), two processes (patient care *n* = 8 and support needed *n* = 2) and one outcome (outcome of the care processes *n* = 3).

In Ethiopia, Uganda and Zimbabwe, 34 pharmacy practice indicators were developed using literature and policy reviews.^42^ The indicators included five domains: system (*n* = 5), storage (*n* = 7), services (*n* = 6), dispensing (*n* = 8) and rational drug use (*n* = 8). To test the functionality of the set in pharmacy practice settings, data from pharmacies were collected using direct observation, record reviews, interviews and simulated clients’ visits. Surveyors were trained on using a survey manual and a data collection sheet which included indicator definitions and sampling methodology. Results were presented using histograms and spider charts showing an assessed score against an ‘ideal’ Good Pharmacy Practice Score.

In the Netherlands,^28^ an initial set of 159 indicators was generated from a literature review, pharmacy practice guidelines, prescribing guidelines and pharmacy‐related indicators from other initiatives. Two consensus rounds followed: round one included pharmacy practice experts, and round two included practising pharmacists. During the consensus process, the main criteria for inclusion were relevance for pharmacy practice and validity for quality of care or risk of harm to patients. Clarity of wording, feasibility of data collection and qualitative comments were also examined. To further test the feasibility of data collection, a field test was conducted in which participating pharmacies were asked to provide data on the proposed indicators. The process resulted in a modified set of 42 indicators including: patient counselling (*n* = 6), clinical risk management (*n* = 10), compounding (*n* = 7), dispensing (*n* = 3), monitoring of medication use (*n* = 11), quality management (*n* = 5), structure (*n* = 13), process (*n* = 18) and outcome indicators (*n* = 11).

A second study in the Netherlands^29^ involved the development of a set of 52 quality indicators by the Dutch Health Care Transparency Programme. The majority of these indicators originated from the set of indicators generated in the previous study.^28^ The additional QIs related to patient counselling and safety, and health insurance companies. The indicators covered 10 domains: continuity of pharmaceutical care (*n* = 4), patient counselling (*n* = 3), clinical risk management (*n* = 12), compounding (*n* = 3), dispensing (*n* = 6), monitoring of medication use (*n* = 10), self‐care support and over‐the‐counter medications (*n* = 2), logistics (*n* = 5), quality management (*n* = 6) and professional development (*n* = 1). These indicators represented 21, 19 and 12 structure, process and outcome indicators, respectively. To assess the validity of the indicators, an expert panel applied a comprehensive ‘Indicator Assessment Framework’ to the data on the QI set collected from 1807 Dutch community pharmacies in 2011. The framework included examination of the indicators’ content validity, absence of selection bias, absence of measurement bias and statistical reliability. For the first three criteria, an expert panel rated the indicators as follows: meeting the requirements, partly meeting the requirements or not meeting the requirements. The expert panel considered pharmacists’ comments, questions and response rate. Statistical reliability was assessed for numerical indicators only. Of 52 quality indicators, 13 met all four criteria, 12 were structure indicators, and one was a process indicator.

In the UK,^31^ focus groups were conducted to develop a conceptual framework characterising healthcare quality in the community pharmacy setting. The study included patients and their carers, pharmacists and pharmacy staff, and National Health Service staff who commissioned pharmacy services. A constant comparative iterative analysis was used to interpret the data followed by identification of themes and domains that used to build the conceptual framework. Three dimensions emerged: accessibility, effectiveness and positive perceptions of the experience with each dimension associated with structure, process and outcome domains. The structure domains (*n* = 8) included premises, equipment, technology, information and data, patient information, medicines, services, pharmacy team (skills and numbers), communication systems, management, professionalism, internal quality systems and financial resources. Processes (*n* = 2) included providing standardised care and providing individualised care. Finally, outcome domains consisted of patient‐specific outcomes, pharmacy‐specific outcomes, societal outcomes and health status.

In the United States,^33^ a literature review and four focus groups with patients were conducted to identify quality attributes of pharmacy services and develop a consumer assessment survey. These attributes were developed into survey items. Patients’ ability to navigate the survey was evaluated using interviews with 12 pharmacy users. An additional evaluation was conducted with pharmacy professors, as well as Pharmacy Quality Alliance and practising pharmacists. The process produced a 50‐item pilot survey. The survey items were assessed based on confirmatory factor analysis, frequency of missing data and standard psychometric methods: variability, reliability and validity. The internal consistency was calculated using Cronbach's coefficient alpha, whereas the reliability within the pharmacy level was tested by using the analysis of variance. Construct validity was assessed by measuring the association between each survey item and its hypothesised composite measure. In terms of variability, the survey results were tested for ceiling effects (i.e. when the scale does not allow people to report higher levels of quality) and floor effects (when scale does not allow people to report lower levels of quality). The assessment resulted in three global indicators (overall pharmacy services, overall pharmacy staff and overall information about medication), and 15 items summarised in three multi‐items domains: general staff communication, health and medication‐focused communication and clarity of written information about medication.

In Thailand,^35^, ^36^ the reliability and feasibility of a 40‐item tool for quality assessment in community pharmacies were evaluated. The tool was originally developed by the Community Pharmacy Association with technical support from the FIP. The 40 items comprised five domains: premises and facilities (*n* = 7), personnel (*n* = 5), drug inventory and stocking (*n* = 7), dispensing and patient care (n = 17), and patient satisfaction and health promotion (*n* = 4). Data from interviews with pharmacists and observational methods were used to score quality indicators. The reliability of the quality indicator tool was tested by the Cronbach’s alpha coefficient and showed good reliability (0.87). Similarly, feasibility was evaluated by score analysis. The results were plotted by histograms of each domain’s assessable scores against its possible maximum score and were used to reflect how well the quality questions in that domain could be answered.

In a second study in the UK,^43^ multiple methods were used to derive quality indicators. A postal questionnaire was sent to community pharmacists and dispensing doctors to identify services provided and monitoring systems in place to record services. This was followed by in‐depth case studies in community pharmacies and dispensing doctor practices. The results were thematically analysed, and quality characteristics were identified that related to service provision. In the next stage,^32^ a two‐round Delphi‐type survey of the identified characteristics was sent to participants representing three stakeholder groups: dispensing doctors, community pharmacists and patients/lay members. Participants confirmed and ranked the importance of characteristics based on representing good quality criterion. The process produced 23 characteristics of good quality pharmaceutical service and covered four broad categories: patient safety and dispensing, patient–provider interaction, workplace culture and public health.

Pharmacy claim data for dispensed prescriptions have also been used to measure and monitor quality in community pharmacies. For example, in the United States, 22 pharmacies’ claim dispensing data were evaluated to test the performance of community pharmacies.^34^ The set included: proportion of days the patient was dispensed the medication of particular class (*n* = 7), gap in therapy (*n* = 7), diabetes medication dosing (*n* = 3), suboptimal asthma control (*n* = 1), absence of asthma controller (*n* = 1) and use of high‐risk medications in the elderly (*n* = 2). Each pharmacy was required to have a minimum of 30 patients for each measure to be included in the evaluation. Additionally, the measures were required to have sensitivity to variation between pharmacies. Less than 10% of pharmacies were evaluable for all measures except one measure associated with the use of high‐risk drugs in the elderly. This measure and another measure related to medication adherence showed potential for use as performance measures as they demonstrated room for improvement and variation among pharmacies.

In Canada,^40^ the feasibility of using pharmacy claim data to assess four quality indicators was also evaluated. The indicators included two safety indicators (dispensing of contraindicated benzodiazepines to seniors and dispensing of non‐selective beta‐blockers to patients with respiratory disease) and two effectiveness indicators (dispensing asthma or hypertension medications to non‐compliant patients). The proportion of community pharmacies where services were provided frequently (i.e. dispensed the relevant medication five or more times over the 1‐year period) was required to enable the reliable assessment of performance indicators. The study found that 86% of pharmacies provided sufficient services to assess performance on all four indicators. The study identified pharmacies that performed well, as well as pharmacies that needed to improve.

### Evaluating the effects of quality indicators on improving the quality of pharmaceutical care

Four studies explored the effect of quality indicators on quality in community pharmacies over time (Table 2).

**Table 2:** Studies reporting evaluating the effects of quality indicators on improving the quality of pharmaceutical care

First author Country Year	Study design	Participants
Teichert^30^ Netherlands 2016	Two evaluations: Online survey on the QI scores from April 2012 to May 2013; National survey during the whole study period of 5 years for indicators that remained unchanged during the study period	88% (*n* = 1,739) of all community pharmacies in the Netherlands in 2013
Castro^41^ Brazil 2014	Retrospective, longitudinal survey	Workshops held in 2007/2008 and other two evaluations, 2002/2003 and 2010/2011
Pascual^38^ Spain 2010	Assessment of whether the implementation of a quality management system based on the ISO 9001: 2008 standard in the community pharmacy improved processes related to pharmaceutical management and care, as well as client/patient satisfaction.	Community pharmacies selected based upon their implementation of the quality management system
Benrimoj^39^ Australia 2008	Randomly selected pharmacies were coached on the implementation of the standards. Pre‐ and post‐ measurements of the level of adherence to the standards were assessed using pseudo‐patron or simulated patient visits.	50% (*n* = 2,706) of all Australian pharmacies

In the Netherlands,^30^ the quality of pharmaceutical care in community pharmacies has been evaluated annually since 2008. In 2013, two evaluations were conducted. The first investigated improvement in areas assessed by 10 indicators that remained unchanged from 2008–2012 (management, patient experience, audit meetings, protocols for contraindications, medications reviews and five clinical QIs). The second evaluation was to assess changes in quality during the year 2012–2013 in community pharmacies using a set of 66 indicators. The set was developed by all major stakeholders including: community pharmacies, healthcare inspectorate, representatives of patient and consumer organisations and insurance companies. The 66 indicators contained 10 categories: ‘quality management’, ‘continuity of care’, ‘communication with the patient’, ‘clinical risk management’, ‘compounding’, ‘dispensing’, ‘follow up of pharmacotherapy guidelines’, ‘counselling’, ‘logistics’ and ‘training of pharmaceutical staff’. Scores were expressed either as categorical variables (yes/no) or as numerical variables (either a number or a proportion). Multi‐level analysis was used to assess the consistency of scores within each pharmacy for over 5 years. The results demonstrated that scores for structure indicators were higher compared with process and outcome indicators. Overall, scores improved from 2008 to 2013.

In Brazil,^41^ an evaluation of the effect of restructuring a pharmaceutical services system was conducted by applying the indicators of the Self‐Assessment Instrument for Pharmaceutical Services Planning. The instrument consisted of indicators in aspects of management, essential medicines selection, stock, storage, distribution, transport, prescription medication, dispensing, human resources and pharmacovigilance. The evaluation was conducted over three time periods (2002–2003, 2007–2008 and 2011–2012). For each indicator, quality was scored from one to three, with three is indicating the best quality. The results showed that the introduction of strategies to monitor quality led to improved management practice. Less satisfactory results were observed with prescribing and dispensing.

In Spain,^38^ a study was conducted to investigate the effects of the implementation of a quality management system (based on the international standard ISO 9001: 2008) in the community pharmacy. Sixteen process indicators were studied over time (the value of all pharmacies was averaged, and the time evolution of each indicator was adjusted to a straight line). The 16 indicators were related to internal management (*n* = 5), pharmaceutical care (*n* = 9) and customer/patient satisfaction (*n* = 2). Improvement was demonstrated in 10 of the 16 indicators including symptom improvement and patients who received health education.

In Australia,^39^ a quality improvement package was implemented in relation to the provision of non‐prescription medicines. The package included four standards with 20 criteria including: resource management (*n* = 3), customer care and advice (supply (*n* = 6), indirect supply (*n* = 2) and documentation (*n* = 3)), pharmacy design and environment (*n* = 3), and rights and needs of customers (*n* = 3). Half of all Australian pharmacies were randomly selected and included in the study. Each pharmacy was audited on the use of standards of practice for the provision of non‐prescription medicines. Three visits were conducted 7 weeks apart. During these visits, an assessment of the pharmacy's level of compliance and pseudo‐patron visits were used to monitor quality. After two visits, more than 80% of pharmacies had met most criteria. The lowest level of compliance was for indicators related to the documentation process. In visit three, there was a significant improvement compared with visits one and two. The results showed that pharmacies had low levels of compliance with written operating procedures but these improved over time.

## Discussion

### Main findings

Few studies have evaluated the effects of quality indicators on improving the quality of pharmaceutical care. This review identified 15 studies from 12 countries (and six continents) and reported a variety of methods for the development of quality indicators in the community pharmacy setting. Few studies included psychometric testing to assess the suitability of quality indicators.

### Strengths and limitations

Duplicate independent screening of the search results minimised the risk of bias and omission. Bias was further reduced by having no language or country limitations, and this is also likely to have increased the generalisability of the results. Due to limited resources, duplicate data extraction was not undertaken. However, duplicate extraction is not an obligatory item in conducting a scoping review according to PRISMA‐ScR.^27^ The review focused on the methods involved in developing and evaluating quality indicators and not the specific indicators. Professional translators were not used for non‐English records.

### General discussion

Quality indicators are often constructed using consensus methods combined with available published evidence or literature reviews. This is probably because scientific evidence in health care is limited or not methodologically rigorous (e.g. trial based).^44^

Stakeholder involvement varied substantially across the studies (Table 1). Patients, commissioners, general practitioners, public health organisations and insurance companies are all potential stakeholders of community pharmacy services. The involvement of commissioners or insurance companies is relevant especially in developing appropriate indicators that are included in pay‐for‐performance schemes.^30^ This scoping review showed little involvement of public or patient groups in the initial development of indicators. In one study, only pharmacists were included in an expert panel to evaluate the validity and the reliability of a set of quality indicators.^29^ This was justified by stating a qualified assessment was needed from experienced personnel involved in daily community pharmacy practice. In another study, professionals versus other stakeholders were involved in the development of QIs for cardiovascular risk management.^45^ The study reported that the professionals were ‘more qualified in assessing these QIs’ than other possible stakeholders. Patient values, preferences and characteristics in terms of quality should also be explored as these have been shown to have a positive impact on knowledge and medication adherence.^46^, ^47^

In the UK, over £1 billion is spent annually on the quality and outcome framework for general practices.^48^ This level of investment includes testing and piloting, and protocols have been developed for this purpose.^48^ The adoption of a protocol‐based approach to the development of quality indicators for community pharmacy could assist in the production of valid and reliable outputs.

The quality indicators identified in these studies broadly covered evaluating aspects of pharmacy design and environment, management, personnel, workplace culture, public health and promotion, medicine stock levels, delivery and refill, storage, patient care, patient counselling, over‐the‐counter medications, safety, compounding, dispensing, pharmacovigilance and professional development. Other aspects were less common in terms of quality management (e.g. errors and complaint management, patient experience and adverse drug reactions) and clinical risk management (e.g. the percentage of patients who concurrently use oral anticoagulants and co‐trimoxazole, percentage of patients with documented contraindication of heart failure who are dispensed NSAIDs). Few studies presented indicators to reflect the Donabedian framework (structure, process and outcome).^22^, ^23^ Process and outcome indicators were less likely to meet psychometric testing compared with structure indicators. For example, Bie *et al.* 28 found that developing a measurable indicator for process or outcome was not feasible due to differences in care organisations and the availability of data in community pharmacies. Schoenmakers *et al.* 29 reported a similar finding. Additionally, the difficulties of collecting clinical outcomes arising from the provision of pharmaceutical care to patients often led to use dispensing data as ‘outcome’ indicators (e.g. dispensing of contraindicated benzodiazepines to seniors and dispensing of non‐selective beta‐blockers to patients with respiratory disease).^28^, ^29^, ^30^, ^34^, ^40^ Evidence was lacking regarding the effect of quality indicators on patient outcome.

Quality improvement should reflect the context in which it is undertaken (i.e. context awareness).^6^ The studies included in this review were undertaken in 15 countries and six continents, demonstrating the interest and activity associated with the use of quality indicators at an international level. The context and target of the indicators in each of these studies differed substantially, and as such, the transferability of the results to other countries might be limited. However, this scoping review is the first to synthesise all identifiable data on this topic and provides an international perspective of the use of this approach for quality improvement in the community pharmacy setting.

Priorities change with time, and QIs should be revised to ensure that they reflect change. For example, in the Netherlands,^29^ the original set of QIs was developed for internal purposes to meet quality standards and to measure improvement. When these indicators were evaluated later for external use, that is, for patient awareness and health insurance companies, only 25% of them met all requirements.

Context also differs with the level of health care provided in different countries. For example, in Thailand,^35^ where pharmacies could be accredited and non‐accredited, QIs were developed to monitor quality in both settings and recommended the use of accredited pharmacies. Whereas, in Ethiopia, Uganda and Zimbabwe,^42^ efforts concentrated on developing QIs to assess structural elements including system, storage, services, dispensing and rational drug use.

The use of objective, reliable data is a challenge associated with the quality measurement of community pharmacy practice. Self‐report is common^30^, ^49^ and is likely to be associated with social desirability bias.^50^ Pharmacy dispensing or claims data have been investigated to derive quality indicators and as a method for avoiding self‐assessment.^30^, ^34^, ^40^ These methods are likely to be less expensive and more reliable compared with using on‐site inspections. However, not all aspects of quality can be evaluated using these data (e.g. over‐the‐counter consultations).

### Implications on policy and research

This scoping review showed that there has been limited investigation of the effects of quality indicators on improving the quality of care in community pharmacies. Future research should seek to adopt a multi‐stakeholder approach to the development of QIs and should evaluate the effect of the introduction of QIs on patient outcome. The inclusion of QIs into policy and contractual arrangements should be evidence‐based and reviewed as an ongoing process to reflect the changing context of health care and concepts of quality.

## Conclusions

Despite the growing emphasis on quality improvement in health care, there is limited reporting of the development and evaluation of QIs for community pharmacy practice. The future development of quality indicators should adopt a multi‐stakeholder approach and include testing of the quality indicators’ psychometric properties. Challenges exist with self‐assessment as well as the development of measurable process and outcome indicators. QIs should reflect the dynamic nature of health care and, as such, should be subject to periodic revision. The long‐term effects of QIs on improvement require further evaluation.

## Declarations

### Conflict of interest

The Author(s) declare(s) that they have no conflicts of interest to disclose.

### Funding

This work was supported by MW’s Health Foundation Improvement Science Fellowship.

## Supporting information


**Appendix S1**. Search strategy.

**Appendix S2**. Characteristics of included studies.
