# Supplementary material for: Quality indicators and community pharmacy services: a scoping review
Source: Int J Pharm Pract. 2019 Jul 2;27(6):490–500. doi: 10.1111/ijpp.12561 (PMC6900189; doi:10.1111/ijpp.12561)
Supplement: Supplementary file 1 — Appendix S1. Search strategy. [file IJPP-27-490-s001.docx]

**Appendix 1. Search strategy**

Embase

('health care quality'/mj OR (('quality'/exp OR quality) AND ('indicator'/exp OR indicator))) AND ('pharmacy'/mj OR (('community'/exp OR community) AND pharmac*)) AND [2008-2018]/py

Pubmed

((("Quality of Health Care"[Majr]) OR quality indicator*)) AND (("Pharmacies"[Majr]) OR community pharmac*) AND ("last 10 years"[PDat])
